# A pan-cancer analysis revealed the role of the SLC16 family in cancer

**DOI:** 10.1080/19336950.2021.1965422

**Published:** 2021-08-23

**Authors:** Jun Li, Jiaheng Xie, Dan Wu, Liang Chen, Zetian Gong, Rui Wu, Yiming Hu, Jiangning Zhao, Yetao Xu

**Affiliations:** a Department of Thoracic Surgery, The First Affiliated Hospital to Nanjing Medical University, Nanjing, Jiangsu, China; b Department of Burn and Plastic Surgery, The First Affiliated Hospital of Nanjing Medical University, Nanjing, Jiangsu, China; c Department of Rheumatology and Immunology, Nanjing Drum Tower Hospital, the Affiliated Hospital of Nanjing University Medical School, Nanjing, Jiangsu, China; d Department of General Surgery, Fuyang Hospital Affiliated to Anhui Medical University, Fuyang, Anhui, China; e Department of Digestive Endoscopy, The First Affiliated Hospital of Nanjing Medical University, Nanjing, Jiangsu, China; f College of Pharmacy, Jiangsu Ocean University, Lianyungang, Jiangsu, China; g Department of Obstetrics and Gynecology, The Affiliated Yantai Yuhuangding Hospital of Qingdao University, Yantai, Shandong, China; h Department of Obstetrics and Gynecology, First Affiliated Hospital of Nanjing Medical University, Nanjing, Jiangsu, China

**Keywords:** SLC, transporter, cancer, immunology, bioinformatics

## Abstract

Cancer is one of the serious diseases that endanger human health and bring a heavy burden to world economic development. Although the current targeted therapy and immunotherapy have achieved initial results, the emergence of drug resistance shows that the existing research is far from enough. In recent years, the tumor microenvironment has been found to be an important condition for tumor development and has profound research value. The SLC16 family is a group of monocarboxylic acid transporters involved in cancer metabolism and the formation of the tumor microenvironment. However, there have been no generalized cancer studies in the SLC16 family. In this study, we conducted a pan-cancer analysis of the SLC16 family. The results showed that multiple members of the SLC16 family could be used as prognostic indicators for many tumors, and were associated with immune invasion and tumor stem cells. Therefore, the SLC16 family has extensive exploration value in the future.

Cancer is still one of the serious diseases endangering human health and has brought a heavy burden to world economic development [1]. At present, the incidence of cancer is still high because of the increase in population, the acceleration of aging, and the bad lifestyle [2]. Early detection, early diagnosis, and early treatment have become the goals of current cancer management [3]. Therefore, in the era of precision medicine, finding effective biomarkers of cancer and exploring their role can promote the development of cancer treatment.

Metabolic change is one of the main hallmarks of cancer [4]. Otto Warburg was a pioneer in the study of cancer metabolism [5]. In the 1920s, Otto Warburg found that tumor cells consumed ten times as much sugar as would be expected for aerobic respiration, which led him to wonder if there was another way of metabolizing sugar in tumors [6]. He came up with the classic antithesis of the Pasteur effect, the “Warburg effect”, in which cancer cells still prefer glycolysis in aerobic conditions, which has greatly improved cancer biology [7]. However, due to the complicated mechanism of tumorigenesis, only the study of aerobic glycolysis alone is not enough. We also need to explore the crosstalk of aerobic glycolysis and other pathways [8]. At present, many products of aerobic glycolysis, such as lactic acid, have been proposed by many scholars to play an important role in the cancer microenvironment and may be the key molecules of aerobic glycolysis and other signaling pathways for crosstalk [8]. These products are mainly transported across the membrane by solute carrier transporters [9]. Therefore, the solute carrier family may be a participant in tumorigenesis and a potential target for future tumor therapy.

The SLC16 family consists of 14 members, namely SLC16A1-A14 [10]. Research on the SLC16 family began in the 1970s when researchers observed that the transport of L-lactic acid was specifically inhibited by α-Cyano-4-Hydroxy-Cinnamate (CHC) [11]. This suggests that there is a specific lactate transporter in the cell. Since then, other homologs have gradually been discovered, making up the existing SLC16 family [12]. Due to the metabolic reprogramming properties of cancer, this group of monocarboxylic acid transporters (MCTs) plays an important role in cancer metabolism [13].

The nomenclature of solute transporters is different from that of monocarboxylic acid transporters. The former is named according to the chronological sequence of gene sequencing, while the latter is named according to transporter function [11]. MCT1-4 are the most intensively studied transporters, which are closely related to the transport of glycolysis products (lactic acid, pyruvate, ketone bodies) [12]. Although MCT 5–14 have not been systematically studied, some new developments in recent years have revealed their significance in human physiology and pathophysiology. Structurally, there is a high degree of homology among MCTs [9]. All MCTs contain 12 transmembrane domains and intracellular N-terminal and C-terminal. However, despite the highly conserved structure of MCTs, genetic differences and amino acid variability give them some specificity [9]. This is the basis of their specificity in transport function. Moreover, the expression of MCTs is also specific to tissues and cells [13].

It is because of the important role of MCTs in cell homeostasis regulation that they have extensive exploration value in human physiology and pathophysiology. MCT1-4(SLC16A1, SLC16A7, SLC16A8, SLC16A3) is mainly involved in the transport of monocarboxylic acids such as lactic acid and is involved in the maintenance of cell pH value, thus maintaining homeostasis [14]. Abnormal expression of MCT1-4 can cause a series of functional disorders. MCT1, MCT2, and MCT4 are expressed in a variety of tissues such as liver, kidney, and skin, especially MCT1 is the most widely expressed [15]. However, MCT3 was mainly expressed in retinal cells [16]. In cancer cells, their expression is thus up-regulated due to high levels of glycolysis [17]. Cancer drugs targeting MCT1-4 are also being developed, such as AZD3965 for lymphoma [18]. In addition, their mutations have been linked to benign diseases, such as the association between MCT1 and hyperinsulinemia and obesity [19,20]. MCT5(SLC16A4) is mainly expressed in the liver, kidney [12]. MCT5 is also expressed in the basolateral membrane of the colon and may be involved in nutrient absorption [21]. Lin et al. found that MCT5 expression was up-regulated in colon cancer, revealing its potential significance in colon cancer [22]. MCT6(SLC16A5) is found expressed in the small intestine [23]. Recent studies have found that MCT6 may act as a drug transporter in humans, mediating the absorption of a variety of drugs such as bumetanide in the small intestine [23]. Some scholars have also found that MCT6 is also associated with ototoxicity caused by cisplatin chemotherapy [12]. MCT7(SLC16A6) is mainly expressed in the liver and kidney and is involved in the transport of ketone bodies. Its mutation may lead to metabolic disorders of ketone bodies and triglycerides [24]. Recent research has found that MCT7 may be linked to human height [25]. MCT8(SLC16A2) is expressed in a variety of tissues but is most significant in the thyroid [26]. Mutations in MCT8, which mediates thyroxine transport, lead to a rare disease called Allen-Herden-Dudley syndrome [26]. This syndrome is caused by mutations in MCT8 that lead to impaired transmembrane transport of thyroid hormone across the brain barrier. MCT10 is a transporter similar in function and structure to MCT8, which mainly mediates the transport of aromatic amino acids and is highly expressed in the liver and kidney [27]. Mutations in MCT10 may be associated with nonalcoholic steatohepatitis [28]. MCTs11-13 was highly expressed in the liver and kidney [29]. Existing studies have confirmed that MCT11(SLC16A11) and MCT13(SLC16A13) are related to type 2 diabetes, and MCT12(SLC16A12) is involved in the development of juvenile cataracts [30,31]. There are few related studies on MCT14(SLC16A14), and it is found to be expressed in the peripheral nervous system, which may be related to ethanol transport [32]. Some other studies have found that MCT14 is expressed in renal tubules and mediates phosphate transport, but specific studies are still limited [33]. At present, there are few studies on MCT9 (SLC16A9).

However, there has been no pan-cancer analysis of the SLC16 family. In our study, we performed a pan-cancer analysis of the SLC16 family in a variety of cancers, including expression analysis, survival analysis, immune correlation analysis, etc. Our results can provide references for further research on the SLC16 family in the future.

## Methods

### Data download

From the Xena Browser website(https://xenabrowser.net/datapages/), we downloaded data needed for analysis, including RNA-Seq, clinical data (Phenotype and survival data), Immune subtypes, DNA methylation induced tumor stem cell properties (DNAss), and mRNAs induced tumor stem cell properties (RNAss) data. The data of 33 cancer types were obtained, including Acute Myeloid Leukemia(LAML, 151 samples), Adrenocortical carcinoma(ACC, 79 samples), Cholangio carcinoma(CHOL, 45 samples), Bladder Urothelial Carcinoma(BLCA, 430 samples), Breast invasive carcinoma(BRCA, 1217 samples), Cervical squamous cell carcinoma and endocervical adenocarcinoma(CESC, 309 samples), Colon adenocarcinoma(COAD, 512 samples), Uterine Corpus Endometrial Carcinoma(UCEC, 583 samples), Esophageal carcinoma(ESCA, 173 samples), Glioblastoma multiforme(GBM, 73 samples), Head and Neck squamous cell carcinoma(HNSC, 546 samples), Kidney Chromophobe(KICH, 89 samples), Kidney renal clear cell carcinoma(KIRC, 607 samples), Kidney renal papillary cell carcinoma(KIRP, 321 samples), Lymphoid Neoplasm Diffuse Large B-cell Lymphoma(DLBC, 48 samples), Liver hepatocellular carcinoma(LIHC, 424 samples), Brain Lower Grade Glioma(LGG, 529 samples), Lung adenocarcinoma(LUAD, 585 samples), Lung squamous cell carcinoma(LUSC, 550 samples), Skin Cutaneous Melanoma(SKCM, 472 samples), Mesothelioma(MESO, 86 samples), Uveal Melanoma(UVM, 80 samples), Ovarian serous cystadenocarcinoma(OV, 379 samples), Pancreatic adenocarcinoma(PAAD, 182 samples), Pheochromocytoma and Paraganglioma(PCPG, 186 samples), Prostate adenocarcinoma(PAD, 551 samples), Rectum adenocarcinoma(READ, 177 samples), Sarcoma(SARC, 265 samples), Stomach adenocarcinoma(STAD, 407 samples), Testicular Germ Cell Tumors(TGCT, 156 samples), Thymoma(THYM, 121 samples), Thyroid carcinoma(THCA, 568 samples), Uterine Carcinosarcoma(USC, 56 samples).

### Expression analysis

First, we analyzed the expression dispersion of the SLC16 family in pan-cancer. The box plot of the SLC16 family expression can reflect the difference of gene expression dispersion. Then, Spearman-correlation analysis was used to calculate the expression correlation between SLC16 family members, and Cytoscape was used for visualization. Next, we used the “PHEATMAP” function to construct a heat map of SLC family expression in a variety of cancers. Finally, tumors with normal controls greater than 5 were screened from the database, and the paired t-test was used to study the expression profile of SLC16A1-A14 in these cancers.

### Survival analysis

To investigate the prognostic value of the SLC16 family in pan-cancer, we extracted patient data with detailed follow-up and survival information. Then, we used the “ggplot2” package to plot the KM survival curves of the SLC16 family in multiple cancers, where P < 0.05 was shown.

Then, univariate Cox regression analysis was further used to identify the prognostic value of each member of the SLC16 family in pan-cancer.

### Tumor microenvironment analysis

UCSC classifies cancer into six immune subtypes (C1, C2, C3, C4, C5, and C6). C1 is the wound healing type, C2 is the dominant type of IFN-γ, C3 is the inflammatory type, C4 is the lymphocyte depleted type, C5 is the immunologically quiet type, and C6 is the TGF-β dominant type. Different immune subtypes have different immune responses, so it is necessary to explore the expression of the SLC16 family in different immune subtypes. Different immune subtypes have different immune responses, so it is necessary to explore the expression of the SLC16 family in different immune subtypes. We use TISIDB (http://cis.hku.hk/TISIDB/index.php) to analyze SLC16A1-A14 expression in the different immune subtypes. Then, the levels of stromal cells and immune cell infiltration in the tumor microenvironment were analyzed by the R software, according to the immune score and stromal score of the transcriptome expression matrix. Finally, the Spearman-correlation analysis was used to further analyze the correlation between mRNA expression of the SLC16 family and tumor stem cell properties.

## Results

### Box Plot revealed the central trend of the SLC16 family in the dataset

**Figure 1.**
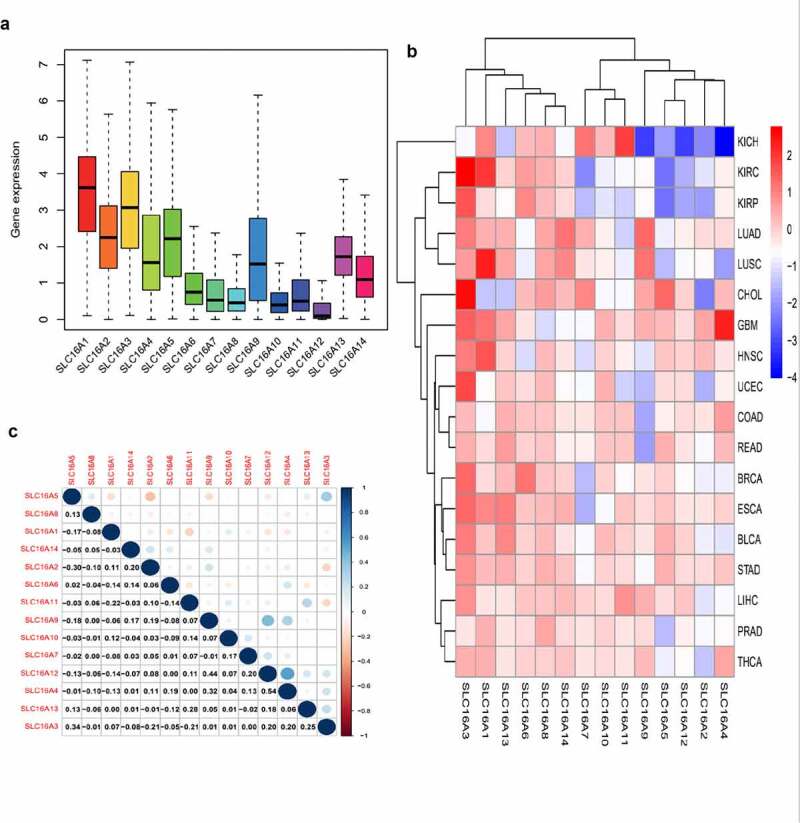
Expression levels of SLC16 family in cancerous and adjacent normal tissues. (a)The box plot showed the fluctuation of SLC16 family expression in cancer: Overall, the expression of SLC16A1 was the highest, and the expression of SLC16A12 was the lowest. The SLC16 family expression has a low dispersion in the data set, which is of great research value. (b)The heat map of the expression of the SLC16 family in each cancer compared to normal tissue: red represents upregulated expression, blue represents downregulated expression, and the shade of the color represents the degree of difference. Acute Myeloid Leukemia(LAML), Adrenocortical carcinoma(ACC), Cholangio carcinoma(CHOL), Bladder Urothelial Carcinoma(BLCA), Breast invasive carcinoma(BRCA), Cervical squamous cell carcinoma and endocervical adenocarcinoma(CESC), Colon adenocarcinoma(COAD), Uterine Corpus Endometrial Carcinoma(UCEC), Esophageal carcinoma(ESCA), Glioblastoma multiforme(GBM), Head and Neck squamous cell carcinoma(HNSC), Kidney Chromophobe(KICH), Kidney renal clear cell carcinoma(KIRC), Kidney renal papillary cell carcinoma(KIRP), Lymphoid Neoplasm Diffuse Large B-cell Lymphoma(DLBC), Liver hepatocellular carcinoma(LIHC), Brain Lower Grade Glioma(LGG), Lung adenocarcinoma(LUAD), Lung squamous cell carcinoma(LUSC), Skin Cutaneous Melanoma(SKCM), Mesothelioma(MESO), Uveal Melanoma(UVM), Ovarian serous cystadenocarcinoma(OV), Pancreatic adenocarcinoma(PAAD), Pheochromocytoma and Paraganglioma(PCPG), Prostate adenocarcinoma(PAD), Rectum adenocarcinoma(READ), Sarcoma(SARC), Stomach adenocarcinoma(STAD), Testicular Germ Cell Tumors(TGCT), Thymoma(THYM), Thyroid carcinoma(THCA), Uterine Carcinosarcoma(USC). (c) Correlation plot based on Spearman Correlation test results to show the correlation of gene expression among the SLC16 family members across all 33 cancer types: Among them, SLC16A4 and SLC16A12 have the strongest correlation. The correlation value was 0.54

The box plot shows the fluctuations in the SLC16 family expression in the data (Figure 1a). The height of the plot represents the discreteness between a set of data. Our study found that the SLC16 family expression has a low dispersion in the data set, which is of great research value.

### The heat map showed the differential expression of the SLC16 family in tumors and normal tissues

From the heat map, we can see the expression of the SLC16 family in pan-cancer compared with normal tissues (Figure 1b). Red represents up-regulated expression, blue represents down-regulated expression, and color shades represent a significant degree of difference.

### Analysis of correlations between members of the SLC16 family in multiple cancers

Through the Spearman correlation test, we can calculate the correlation between each member of the SLC16 family and visualize it through R software. The correlation graph shows the correlation values among the members (Figure 1c). This is meaningful for us to understand the SLC16 family’s mode of action.

### Expression of SLC16 family across cancers

**Figure 2.**
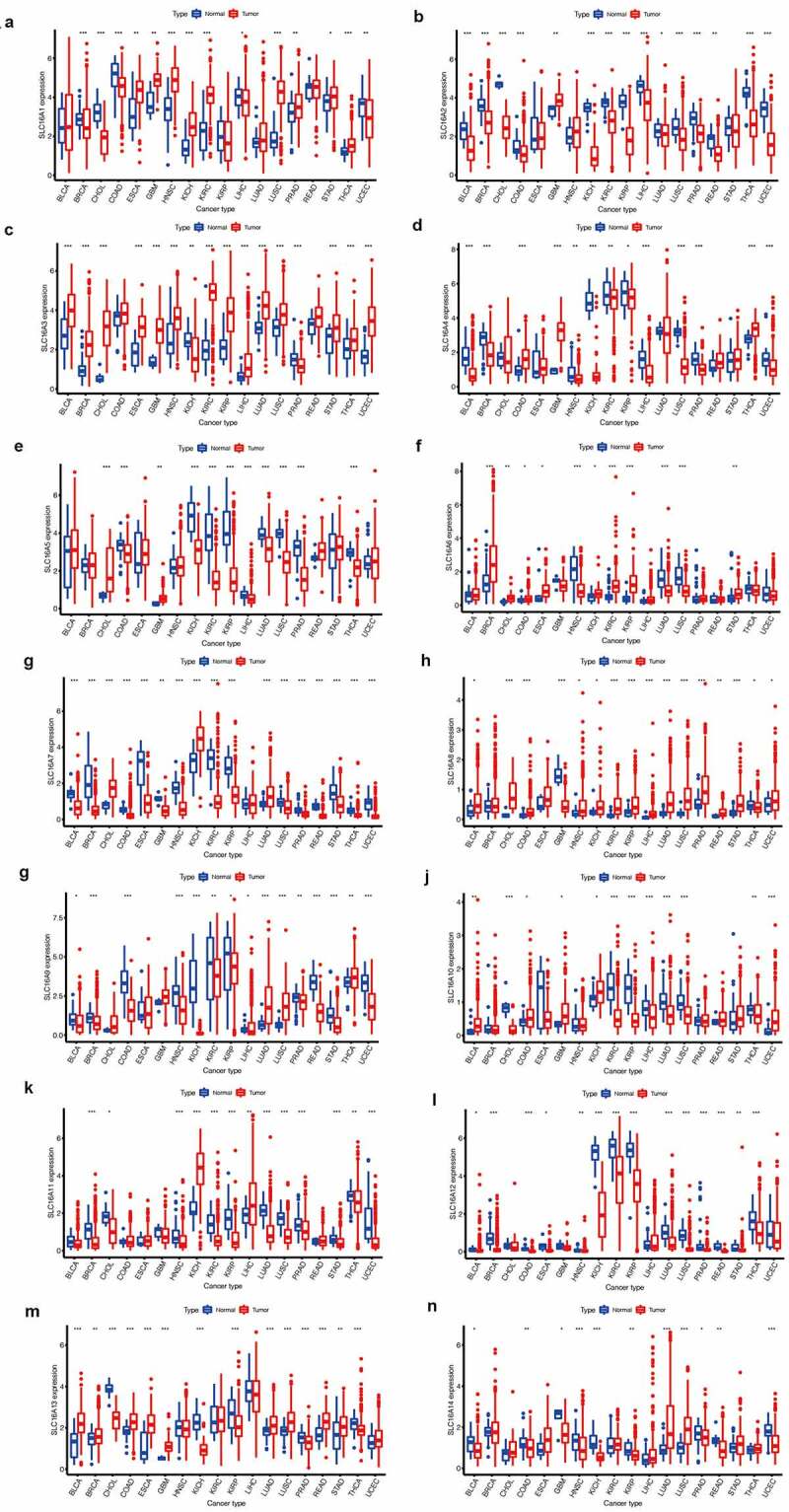
SLC16 family gene expression levels in different cancer types and normal tissue. The red rectangle box represents gene expression levels in tumor tissue and the blue rectangle box represents normal tissue. * P < 0.05, ** P < 0.01, and *** P < 0.001. (a)SLC16A1 is differentially expressed in a variety of cancers, including BRCA, CHOL, COAD, ESCA, GBM, HNSC, KICH, KIRC, LIHC, LUSC, PRAD, STAD, THCA, UCEC. (b)SLC16A2 is differentially expressed in a variety of cancers, including BLCA, BRCA, CHOL, COAD, GBM, KICH, KIRC, KIRP, LIHC, LUAD, LUSC, PRAD, READ, THCA, and UCEC. (c)SLC16A3 is differentially expressed in a variety of cancers, including BLCA, BRCA, CHOL, ESCA, GBM, HNSC, KICH, KIRC, KIRP, LIHC, LUAD, LUSC, PRAD, STAD, THCA, and UCEC. (d)SLC16A4 is differentially expressed in a variety of cancers, including BLCA, BRCA, COAD, GBM, HNSC, KICH, KIRC, KIRP, LIHC, LUSC, PRAD, THCA, and UCEC. (e)SLC16A5 is differentially expressed in a variety of cancers, including CHOL, COAD, GBM, KICH, KIRC, KIRP, LIHC, LUAD, LUSC, PRAD, THCA. (f)SLC16A6 is differentially expressed in a variety of cancers, including BRCA, CHOL, COAD, ESCA, HNSC, KICH, KIRC, KIRP, LUAD, LUSC, and STAD.(g)SLC16A7 is differentially expressed in a variety of cancers, including BLCA, BRCA, CHOL, COAD, ESCA, GBM, HNSC, KICH, KIRC, KIRP, LUAD, LUSC, PRAD, READ, STAD, THCA, and UCEC. (h)SLC16A8 is differentially expressed in a variety of cancers, including BLCA, CHOL, COAD, GBM, HNSC, KICH, KIRC, KIRP, LIHC, LUAD, LUSC, PRAD, READ, STAD, THCA, and UCEC. (i)SLC16A9 is differentially expressed in a variety of cancers, including BLCA, BRCA, COAD, HNSC, KICH, KIRC, KIRP, LIHC, LUAD, LUSC, PRAD, READ, STAD, THCA, and UCEC. (j)SLC16A10 is differentially expressed in a variety of cancers, including BLCA, CHOL, COAD, GBM, KICH, KIRC, KIRP, LIHC, LUAD, LUSC, THCA, and UCEC. (k)SLC16A11 is differentially expressed in a variety of cancers, including BRCA, CHOL, HNSC, KICH, KIRC, KIRP, LIHC, LUAD, LUSC, LUAD, LUSC, PRAD, STAD, THCA, UCEC. (l)SLC16A12 is differentially expressed in a variety of cancers, including BLCA, BRCA, COAD, RSCA, HNSC, KICH, KIRC, KIRP, LUAD, LUSC, PRAD, READ, STAD, and THCA. (m)SLC16A13 is differentially expressed in a variety of cancers, including BLCA, BRCA, CHOL, COAD, ESCA, GBM, KICH, KIRP, LUAD, LUSC, PRAD, READ, STAD, and THCA. (n)SLC16A14 is differentially expressed in a variety of cancers, including BLCA, COAD, GBM, HNSC, KICH, KIRP, LUAD, LUSC, PRAD, READ, and UCEC

To further investigate the significance of the SLC16 family in various tumors, we analyzed the expression of the SLC16 family in different tumors (Figure 2). We found that the SLC16 family was differentially expressed in a variety of cancers, such as BLCA, CHOL, LUAD, etc, suggesting that they may play an important role in cancer and may be genes that promote cancer.

### Survival analysis

**Figure 3.**
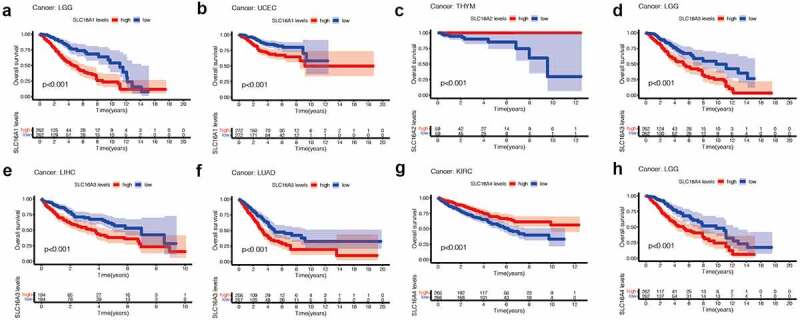
Kaplan-Meier survival curves comparison of high and low expression of SLC16 family members in pan-cancer. Only survival diagrams with P < 0.001 were shown. Other survival analysis diagrams with p < 0.05 were included in the supplementary document. (a)SLC16A1 is a poor prognostic biomarker in Brain Lower Grade Glioma(LGG, 524 samples). (b)SLC16A1 is a poor prognostic biomarker in Uterine Corpus Endometrial Carcinoma(UCEC, 544 samples). (c)SLC16A2 is a good prognostic biomarker in Thymoma(THYM, 118 samples). (d)SLC16A3 is a poor prognostic biomarker in Brain Lower Grade Glioma(LGG, 524 samples). (e)SLC16A3 is a poor prognostic biomarker in Liver hepatocellular carcinoma(LIHC, 368 samples). (f)SLC16A3 is a poor prognostic biomarker in Lung adenocarcinoma(LUAD, 513 samples). (g)SLC16A4 is a good prognostic biomarker in Kidney renal clear cell carcinoma(KIRC, 531 samples). (h)SLC16A4 is a poor prognostic biomarker in Brain Lower Grade Glioma(LGG, 524 samples)

**Figure 4.**
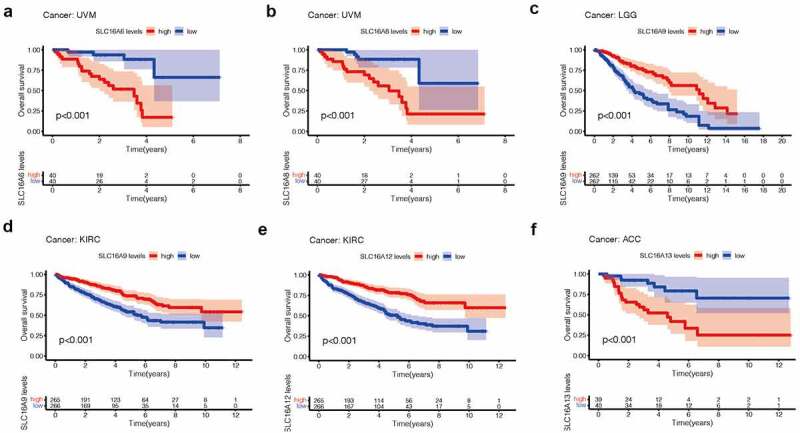
Kaplan-Meier survival curves comparison of high and low expression of SLC16 family members in pan-cancer. Only survival diagrams with P < 0.001 were shown. Other survival analysis diagrams with p < 0.05 were included in the supplementary document. (a)SLC16A6 is a poor prognostic biomarker in Uveal Melanoma(UVM, 80 samples). (b)SLC16A8 is a poor prognostic biomarker in Uveal Melanoma(UVM, 80 samples). (c)SLC16A9 is a good prognostic biomarker in Brain Lower Grade Glioma(LGG, 524 samples). (d)SLC16A9 is a good prognostic biomarker in Kidney renal clear cell carcinoma(KIRC, 531 samples). (e)SLC16A13 is a good prognostic biomarker in Adrenocortical carcinoma(ACC, 79 samples). (f) SLC16A12 is a poor prognostic biomarker in Kidney renal clear cell carcinoma(KIRC, 531 samples)

**Figure 5.**
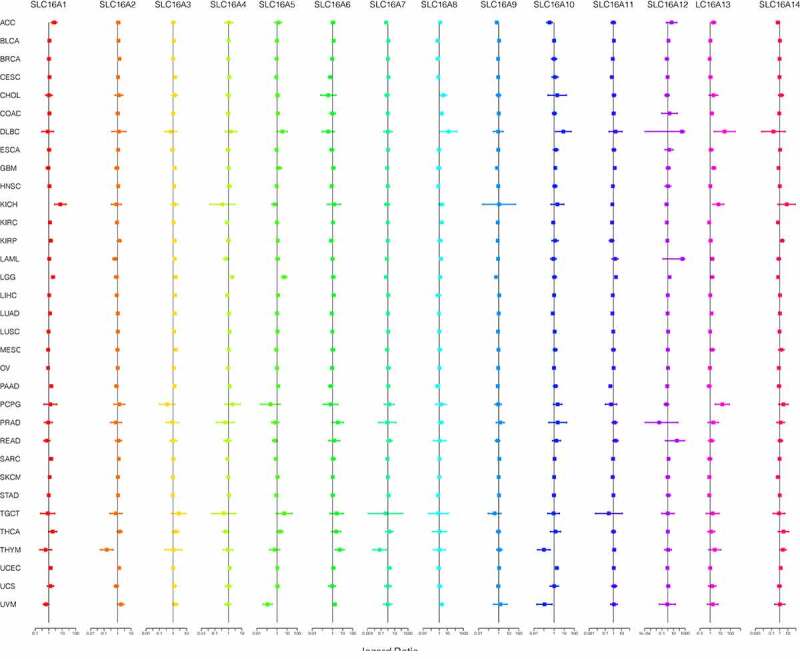
Considering that the survival analysis was influenced by multiple variables of the patient, univariate Cox regression was performed to reduce the influence of other variables. Association of SLC16 family expression with patient overall survival for different cancer types. The forest plots with the hazard ratios and 95% confidence intervals for overall survival for different cancer types to show survival advantage and disadvantage with increased gene expression of the SLC16 family. Univariate Cox proportional hazard regression models were used for the association tests. Hazard ratio <1 represents low risk and hazard ratio >1 represents high risk

We found SLC16 family is associated with the prognosis of various types of cancer (Figure 3, Figure 4, Supplementary Figure 1, Supplementary Figure 2). Survival diagrams with P < 0.001 were shown in Figure 3 and Figure 4. Other survival analysis diagrams with p < 0.05 were included in the Supplementary Figure 1 and Supplementary Figure 2. As is shown above, tumors of prognostic value to members of the SLC16 family and their survival curves are listed. We can see that the SLC16 family is a potent prognostic marker for a variety of tumors. Considering that the survival analysis was influenced by multiple variables of the patient, univariate Cox regression was performed to reduce the influence of other variables. The results also showed that the SLC16 family is a prognostic marker for a variety of cancers (Figure 5).

### Tumor microenvironment analysis

**Figure 6.**
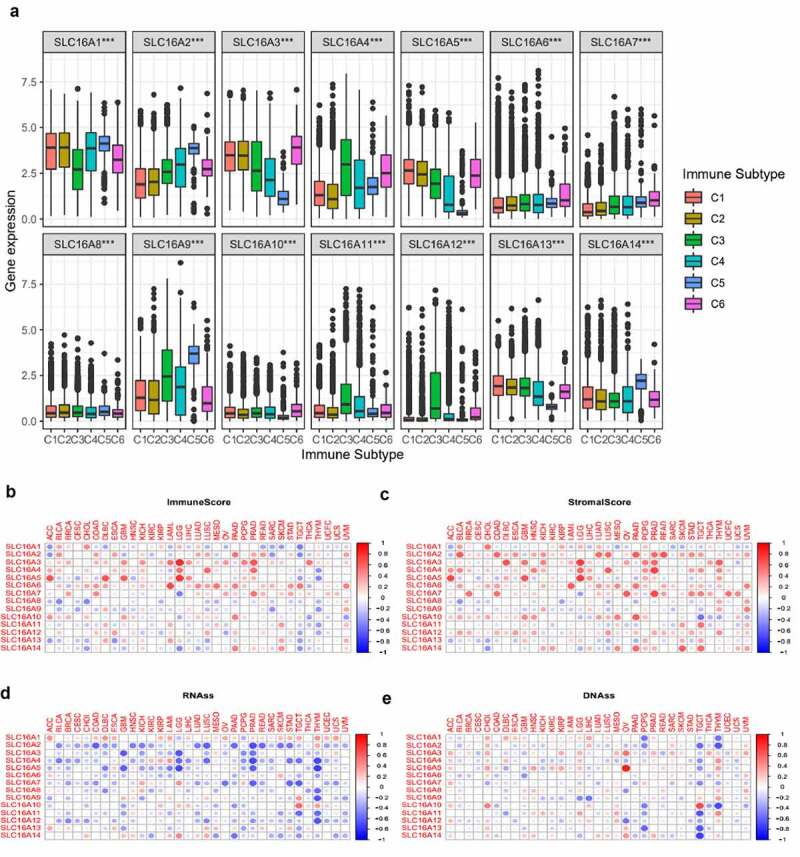
(a)The expressions of SLC16A1-14 were different in different immune subtypes (C1, C2, C3, C4, C5, and C6). C1 is the wound healing type, C2 is the dominant type of IFN-γ, C3 is the inflammatory type, C4 is the lymphocyte depleted type, C5 is the immunologically quiet type, and C6 is the TGF-β dominant type. This suggests that SLC16 family proteins may be related to tumor immunity. (b)Correlation matrix plots to show the association between SLC16 family expression and immune scores of 33 different cancer types based on the ESTIMATE algorithm. Spearman correlation was used for testing. The size of the dots stands for the absolute value of the correlation coefficients. The bigger the size is, the higher the correlation is (higher absolute correlation coefficient). (c)Correlation matrix plots to show the association between SLC16 family expression and stromal scores of 33 different cancer types based on the ESTIMATE algorithm. Spearman correlation was used for testing. The size of the dots stands for the absolute value of the correlation coefficients. The bigger the size is, the higher the correlation is (higher absolute correlation coefficient).(d, e)Association of SLC16 family expression with tumor stemness in 33 different cancer types. Correlation matrix between SLC16 family expression and mRNAs induced tumor stem cell properties (RNAss, Figure 6d) and DNA methylation induced tumor stem cell properties (DNAss, Figure 6e) respectively based on Spearman correlation tests

We found that the expressions of the SLC16 family were different in different immune subtypes (P < 0.05, Figure 6a), suggesting that the SLC16 family may be related to tumor immunity. We also found a correlation between the SLC16 family and immune score (Figure 6b). Most of the SLC16 proteins showed a negative correlation trend with the immune score in cancer, while SLC3, 4, and 5 showed a positive correlation trend with the immune score in cancer. We also found that the SLC16 family was most positively correlated with stromal scores in cancer (Figure 6c). In addition, SLC is associated with mRNAs induced tumor stem cell properties (RNAss) and DNA methylation induced tumor stem cell properties (DNAss) (Figure 6d,6e).

## Discussion

In 1889, Stephen Paget proposed the “seed-soil” theory of cancer, which means that cancer “seeds” can only occur in a specific “soil”: tumor microenvironment [34]. So far, the interaction between “seed” and “soil” has occurred in tumors through multiple processes, such as initiation, progression, invasion, and metastasis [34]. Over the past 50 years, somatic mutation theory has guided the development of cancer mechanisms and related treatments [35]. At present, relevant studies and treatment programs are mainly based on the characteristics of tumor epithelial cells [36]. The treatments based on mutations in tumor cells are widely available. However, in a significant number of patients with these regimens, resistance develops and tumors relapse [36]. In this setting, cancer treatment hits a bottleneck.

In recent years, the role of the tumor microenvironment and related metabolism has become increasingly evident [37]. In addition to tumor cells, there are blood vessels, extracellular matrix, nonmalignant cells, and signaling molecules in the tumor microenvironment [38]. They form a complex regulatory network that promotes tumor growth [39]. Therapies targeting the tumor microenvironment are promising [39]. In general, the tumor microenvironment is anoxic and acidic, which is related to the metabolic characteristics of the tumor [40]. The Warburg effect (aerobic glycolysis) of tumors is an important feature of tumor metabolism [41]. The metabolites generated by the Warburg effect activate multiple signals through complex mechanisms to promote the occurrence and development of tumors [41]. The SLC16 family mediates the transport of various metabolites in tumors, so they may play an important role in the development of cancer, and treatment based on the SLC16 family is worth exploring.

Many members of the SLC16 family have been described concerning cancer. Watson et al. found that SLC16A1 (MCT1) mediated lactate transport in the tumor microenvironment is associated with immune tolerance in melanoma [42]. Xie et al. further studied the role of SLC16A1 in melanoma and found that the expression of SLC16A1 was up-regulated in metastatic melanoma and was associated with poor prognosis and immune cell infiltration [43]. Sohrabi et al. found that SLC16A2 and SLC16A9 play an important role in breast cancer and are associated with poor prognosis of breast cancer [44]. SLC16A3 is a protein highly associated with hypoxia [45]. Studies have shown that hypoxia-inducible factor-1 (HIF-1) can enhance the transcription of SLC16A3, thereby enhancing the proliferation of colon cancer [45]. A multicenter study found that SLC16A5 gene mutation was associated with adverse reactions to platinum-based drugs in testicular cancer patients, which provided a reference for the mechanism of adverse reactions in testicular cancer [46]. Wei et al. found that upregulation of SLC16A6 affects patient outcomes through ketone body transporter pathways [47]. Studies of SLC16A7 have focused on prostate cancer and are associated with the malignant transformation of prostate cancer cells [48]. Moreover, studies have shown that SLC16A7 is associated with the stem cell characteristics of tumors [49]. Mei et al. found that the expression of SLC16A12 was decreased in renal clear cell carcinoma and was associated with poor prognosis, which has a good prognostic guiding significance [50]. However, studies on SLC16A4, SLC16A10, SLC16A11, SLC16A13, and SLC16A14 in cancer are still lacking.

In this study, first, we found that SLC16 family expression was not highly dispersed in pan-cancer, and there was a significant correlation between multiple members. Then, we analyzed the expression of SLC16A1-14 in pan-cancer and found that they were differentially expressed in a variety of cancers, such as liver cancer and cholangiocarcinoma. Next, we performed a survival analysis of SLC16A1-14 in pan-cancer (> 5 normal controls) and found that the SLC16 family can guide the prognosis of many tumors. Finally, we explored the role of the SLC16 family in the tumor microenvironment and found potential relationships between the SLC16 family and immune subtypes, immune score, stromal score, RNAss, and DNAss.

Many tumors with poor prognoses lack effective prognostic indicators, so finding a new biomarker has a lot of benefits for the treatment of tumors [51]. The SLC16 family has been associated with significant changes in prognosis in a variety of cancers, revealing their significance in guiding the prognosis of cancer patients. Moreover, this suggests that the SLC16 family plays an important role in cancer, which is related to their transport role in cancer metabolism. The SLC16 family’s involvement in monocarboxylic acid transport is an important initiating factor in tumor microenvironment formation.

Genomic instability is common in tumors, which predisposes them to genetic mutations [52]. Tumors often accumulate a large number of genetic mutations, providing many potential targets [53]. However, even when neoantigens were expressed in large numbers, tumor immunity was insufficient to kill tumor cells due to tumor-induced immune reediting [54]. How to activate the immune system to treat tumors is a hot topic in oncology research. The FDA has approved immune checkpoint inhibitors to treat a variety of tumors [55]. Therefore, it is of great value to study tumor immunity and identify potential targets. Cancer can be divided into different immune subtypes based on different immune characteristics, and we found that the SLC16 family is differentially expressed in many immune subtypes, which is helpful to understand its role in the immune system [56]. Moreover, we analyzed the relationship between the SLC16 family and immune score or stromal score in the tumor microenvironment. This landscape can provide a reference for us to study the role of SLC16 in the tumor microenvironment.

Currently, the tumor stem cell hypothesis has attracted more and more attention [57]. According to this theory, tumor stem cells in the tumor microenvironment are the source of tumors and an important mechanism for the formation of tumor heterogeneity [57]. Because of their potential role in tumorigenesis and development, tumor stem cell-based therapies hold great promise. We explored the SLC16 family and mRNAs induced tumor stem cell properties (RNAss) and DNA methylation induced tumor stem cell properties (DNAss). The landscape shows the role of the SLC16 family in the characterization of cancer stem cells.

In summary, our study provides a comprehensive overview of SLC16 family expression, prognosis, and their role in the microenvironment. Our results have implications for the understanding and treatment of tumor mechanisms. However, our study is also limited. We lack in vitro and in vivo experiments to verify our results, which will be further improved in the future.

## Conclusion

Members of the SLC16 family are potent biomarkers in cancer and can guide prognosis in many types of cancer. Moreover, members of SLC16 family are associated with immune infiltration and tumor stem cells, which provides new ideas for the exploration of future tumor therapy.

## Supplementary Material

Supplemental Material
