# Supplementary figures and images for: A pan-cancer analysis revealed the role of the SLC16 family in cancer
Source: Channels (Austin). 2021 Aug 23;15(1):528–40. doi: 10.1080/19336950.2021.1965422 (PMC8386723; doi:10.1080/19336950.2021.1965422)

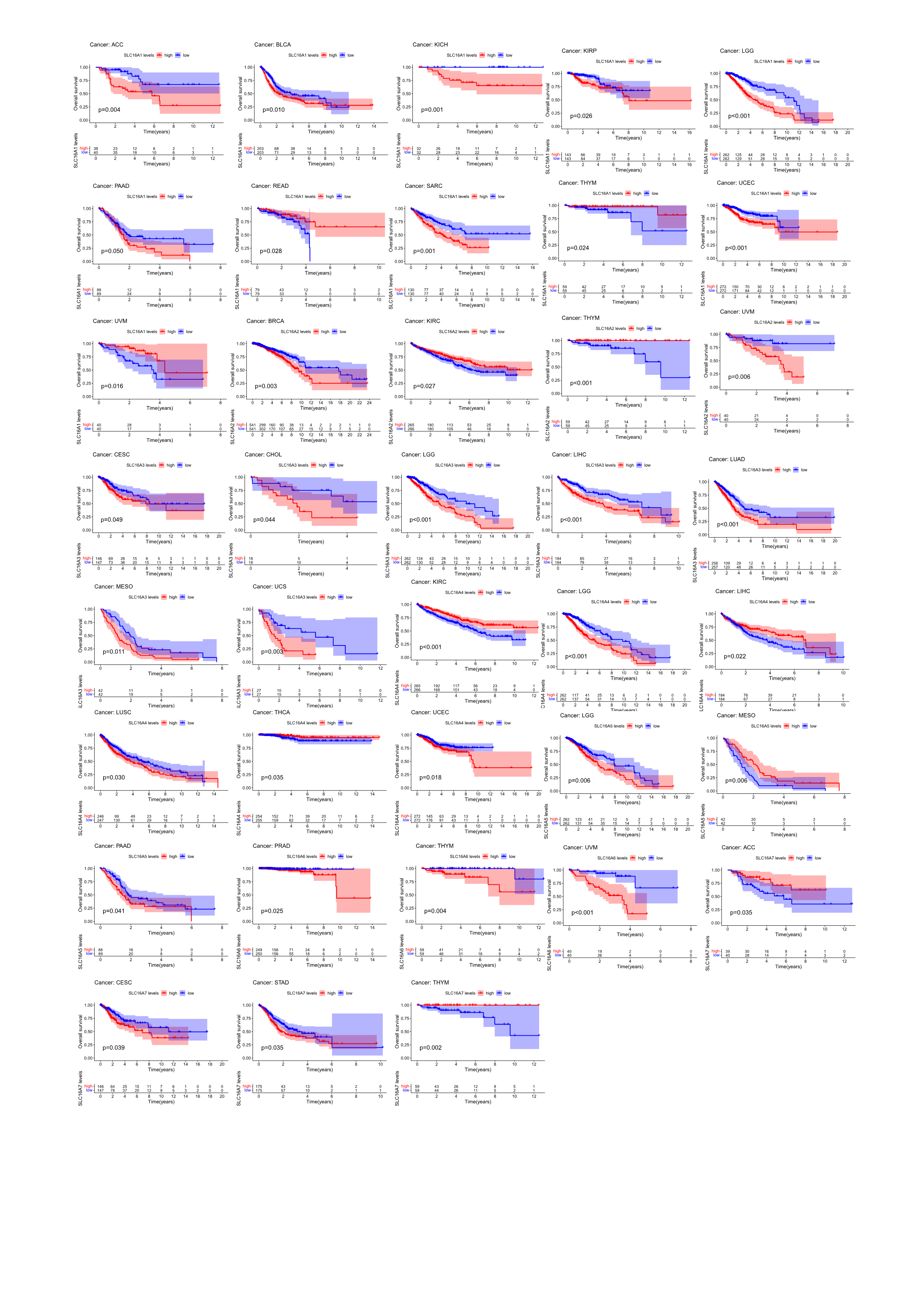

Supplement: Supplemental Material [file KCHL_A_1965422_SM7179.zip › suppl/Figure_S1.png]

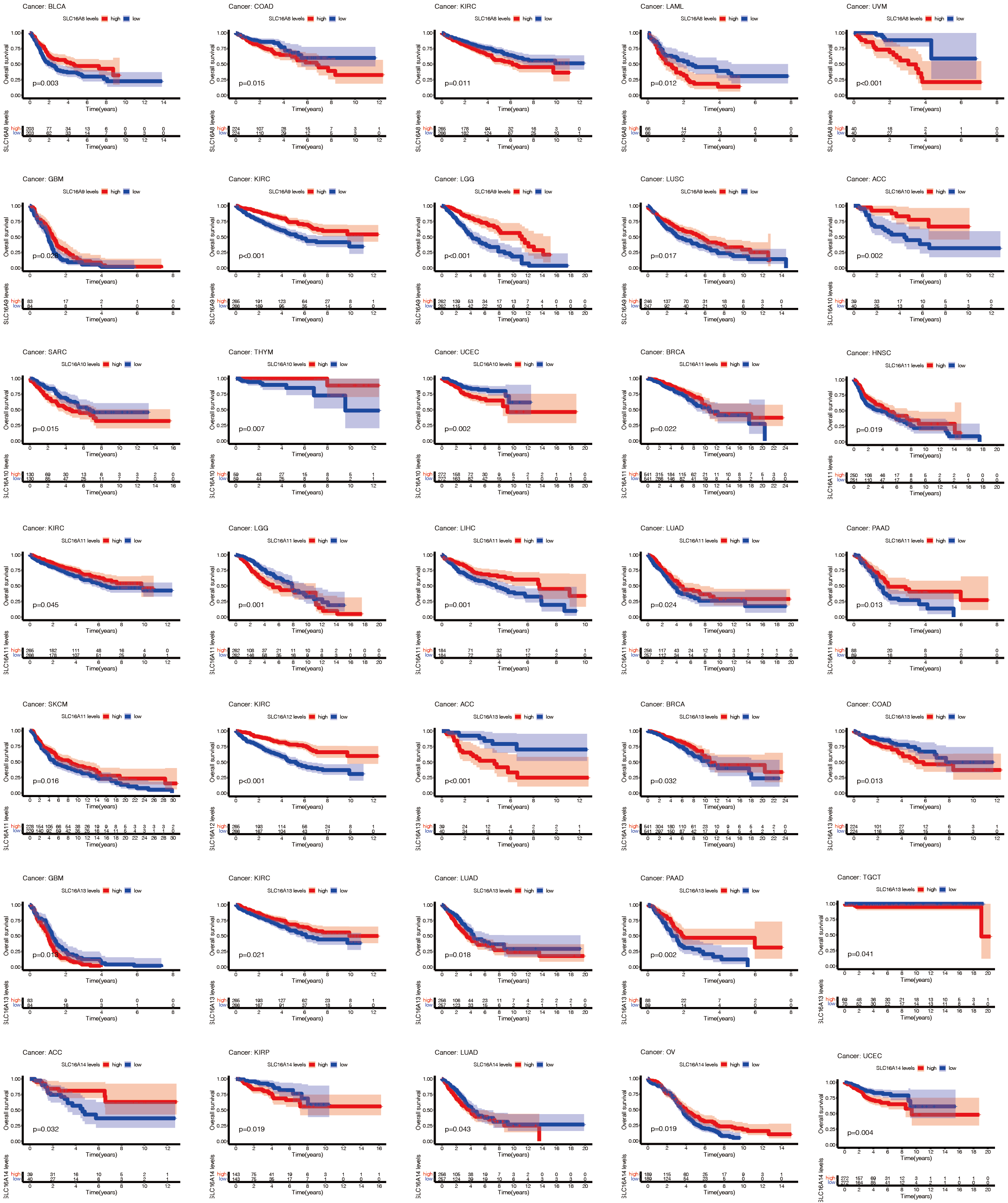

Supplement: Supplemental Material [file KCHL_A_1965422_SM7179.zip › suppl/Figure_S2.png]
